# Zinc Oxide Nanoparticles Cytotoxicity and Release from Newly Formed PMMA–ZnO Nanocomposites Designed for Denture Bases

**DOI:** 10.3390/nano9091318

**Published:** 2019-09-15

**Authors:** Mariusz Cierech, Jacek Wojnarowicz, Adam Kolenda, Agata Krawczyk-Balska, Emilia Prochwicz, Bartosz Woźniak, Witold Łojkowski, Elżbieta Mierzwińska-Nastalska

**Affiliations:** 1 Department of Prosthodontics, Medical University of Warsaw, 02-006 Warsaw, Poland; adam.kolenda@poczta.fm (A.K.); elzbieta.mierzwinska-nastalska@wum.edu.pl (E.M.-N.); 2 Institute of High Pressure Physics, Polish Academy of Sciences, 01-142 Warsaw, Poland; j.wojnarowicz@labnano.pl (J.W.); b.wozniak@labnano.pl (B.W.); w.lojkowski@labnano.pl (W.Ł.); 3 Department of Applied Microbiology, Biological and Chemical Research Centre, Faculty of Biology, University of Warsaw, 02-089 Warsaw, Poland; akra@biol.uw.edu.pl (A.K.-B.); emilia.prochwicz@gmail.com (E.P.)

**Keywords:** denture bases, nanocomposites, poly(methyl methacrylate), zinc oxide nanoparticles (ZnO NPs), cytotoxicity, ZnO NPs release

## Abstract

The goal of the study was to investigate the level of zinc oxide nanoparticles (ZnO NPs) release from polymethyl methacrylate (PMMA)–ZnO nanocomposites (2.5%, 5%, and 7.5% *w*/*w*), as well as from the ZnO NPs layer produced on pure PMMA, and the impact of the achieved final ZnO NPs concentration on cytotoxicity, before the potential use as an alternative material for denture bases. The concentration of ZnO nanoparticles released to the aqueous solution of Zn^2+^ ions was assessed using optical emission spectrometry with inductively coupled plasma (ICP-OES). In the control group (pure PMMA), the released mean for ZnO was 0.074 mg/L and for individual nanocomposites at concentrations of 2.5%, 5%, and 7.5% was 2.281 mg/L, 2.143 mg/L, and 3.512 mg/L, respectively. The median for the ZnO NPs layer produced on PMMA was 4.878 mg/L. In addition, in vitro cytotoxicity of ZnO NPs against the human HeLa cell line was determined through the reduction of 3-(4,5-dimethylthiazol-2-yl)-2,5-diphenyltetrazolium bromide (MTT) dye. The cytotoxicity studies demonstrate that ZnO nanoparticles in the concentrations up to 20 mg/L have no adverse effect on HeLa cells. When compared with the released and cytotoxic concentrations of ZnO NPs, it can be expected that ZnO released from dental prostheses to the oral cavity environment will have no cytotoxic effect on host cells.

## 1. Introduction

The dynamic development in the field of nanotechnology and broadly understood nanoscience open up new application possibilities in the area of consumer products. Many innovative applications of nanotechnology are now available [1]. There is a growing use of nanomaterials in various products, such as cleaning agents, cosmetics, electronics, impregnants, paints, catalysts, and nanocomposites for both construction and medical applications. For most applications of nanomaterials, the study of potential long-term effects on the environment and human health plays a very important role. Specific regulations regarding nanomaterials have been introduced, e.g., the Biocidal Products Regulations (BPR) [2,3]. The definitions contained in the regulation are based on the European Commission’s recommendation concerning the definition of nanomaterials. These provisions apply to active and non-active substances that show the following characteristics; at least 50% of the particles have a size of 1–100 nm in at least one dimension; the particles are in a free state or in the form of an aggregate or agglomerate. Among others zinc oxide nanoparticles (ZnO NPs) have recently gained growing popularity among scientists. ZnO NPs are widely known for their antibacterial properties [4,5,6,7]. At present, numerous tests with the use of ZnO NPs as a contrast medium (imaging), biosensors, anticancer agent, for drug delivery, gene delivery, tissue regeneration, and for diagnostic and therapeutic functions have been carried out [8,9,10,11,12,13,14,15,16,17,18,19,20].

The contemporary development of materials science provides engineers and physicians with the opportunity allowing them not only to develop new materials but also to improve those that have already been tested for years. Polymethyl methacrylate (PMMA) is one of those materials that have been used in dental prosthetics over decades. This is a material whose mechanical properties and effects exerted on oral tissues are well known [21,22]. Today it is difficult to imagine dental prosthodontics without this material. There are only few other possibilities in performance of removable dentures. First of them are flexible materials based on nylon polymers. Nylon is a generic name for certain types of thermoplastic polymers belonging to the class known as polyamides [23]. The second option are cast dentures made of metal alloys (mainly Co-Cr-Mo alloys or titanium alloys) [24]. However, none of them are recommended in cases of severe teeth loss or complete edentulousness.

These considerations prompted the authors of this study to make an attempt to modify the PMMA material to obtain antifungal properties, which are so important in clinical applications. Inflammation of the prosthetic area complicated by a fungal infection is termed as denture stomatitis. It is often observed in patients wearing acrylic extensive dentures who neglect oral and denture hygiene and in favorable local and general factors. The usually applied therapeutic methods cannot be frequently used in prophylaxis of denture stomatitis because of yeast drug resistance. This is another reason why we should look for new solutions in the form of new materials and/or methods for treating this disease. As evidenced in the previous publications [25,26,27], the fabrication of PMMA modified by ZnO NPs is possible in the conditions of an ordinary prosthetic laboratory. PMMA-ZnO composite materials with a filler in the form of nanometric ZnO particles (PMMA-ZnO nanocomposites [28,29]) as a promising material for denture bases are becoming increasingly more popular among scientists and dentists [25,26,27,30,31,32,33,34,35,36,37,38,39,40,41]. A thin layer of saliva is presenting the oral environment between the denture base and oral mucosa. For this reason, only nanoparticles released from the biomaterial can penetrate into the tissue and exert the therapeutic effect. Therefore, in order to achieve therapeutic and preventive effects, the concentration of released ZnO cannot be lower than the minimal inhibitory concentration (MIC) relative to pathogens, but it also cannot be so high as to be able to generate a cytotoxic effect on host cells.

The objective of the study was to characterize zinc oxide release from PMMA–ZnO nanocomposites, as well as from the ZnO NPs layer produced on pure PMMA and the impact of the achieved final ZnO NPs concentration on cytotoxicity, before the potential use as an alternative material for denture bases. The null hypothesis was that no significant difference would be found between pure PMMA and particular modifications with ZnO NPs.

## 2. Materials and Methods

### 2.1. Substrates

The following were used for synthesis and preparation process of ZnO NPs: zinc acetate dihydrate (Zn(CH_3_COO)_2_∙2H_2_O, analytically pure, Chempur, Piekary Śląskie, Poland), ethylene glycol (C_2_H_4_(OH)_2_, pure, Chempur, Piekary Śląskie, Poland), and deionized water (H_2_O) (specific conductance < 0.1 µS/cm, HLP20UV, Hydrolab, Straszyn, Poland). These reagents were used in the condition in which they were received.

### 2.2. Synthesis and Characteristics of ZnO NPs

ZnO NPs were obtained using the microwave solvothermal synthesis according to our original method [25,26,42,43,44,45], which enables precise size control of ZnO NPs (both undoped and doped). The reaction precursor was prepared by dissolving 24.14 g of zinc acetate dihydratein 350 mL of ethylene glycol using the hot-plate magnetic stirrer (70 °C, 450 rpm, SLR, SI Analytics GmbH, Mainz, Germany). After complete dissolution of zinc acetate dihydrate, the solution was poured into a glass bottle with a thread (500 mL, Schott, Mainz, Germany), which was screwed tight and cooled to room temperature (23 °C ± 1 °C). Subsequently, an analysis of water content in the obtained precursor solution (1.12 wt % ± 0.03 wt %) was carried out, and afterwards 1.5551 g of deionized water was added in order to obtain the final water content of 1.49 wt % ± 0.02 wt %.

The reaction driven by microwave radiation was carried out in the MSS2 reactor (3 kW, 2.45 GHz, IHPP PAS, ITeE-PIB, ERTEC-Poland) [46,47]. The parameters of the microwave synthesis were as follows: microwave power—3 kW, pressure—4 bar, heating duration—12 min, volume of Teflon^®^ chamber feedstock—300 mL. The reaction of obtaining ZnO NPs as a result of the reaction of zinc acetate with ethylene glycol is presented in the following general Equation (1):

(1) $${\left({\mathrm{CH}}_{3}\mathrm{COO}\right)}_{2}\mathrm{Zn}+2C_{2}H_{4}{\left(\mathrm{OH}\right)}_{2}\overset{C_{2}H_{4}{\left(\mathrm{OH}\right)}_{2},{\;H}_{2}O,\;T,\;P}{\to }\mathrm{ZnO}\left(↓\right)+H_{2}O+2{\mathrm{CH}}_{3}{\mathrm{COOC}}_{2}H_{4}\mathrm{OH}$$

The effect of the synthesis was suspension of ZnO NPs in ethylene glycol, which was subjected to the sedimentation process. Subsequently, the supernatant was decanted, and the sediment was rinsed with deionized water and centrifuged (MPW-350, MPW Med Instruments, Warsaw, Poland). The rinsing and centrifuging process was repeated four times. The obtained white paste was used for preparing a water suspension of ZnO NPs. A portion of the suspension was rapidly frozen by pouring liquid nitrogen thereon and lyophilised (Lyovac GT-2, SRK System technik GmbH, Riedstadt, Germany) in order to perform analyses of the obtained ZnO NPs.

Water content tests were performed with the use of the titrator (Cou-Lo Aqua MAX KF, GR Scientific, Bedford, UK) operating in line with the assumptions of the Karl Fischer method.

The following parameters were defined for the obtained nanoparticles: skeleton density (ISO 12154:2014, AccuPyc II 1340, Micromeritics^®^, Norcross, GA, USA), specific surface area (SSA) (BET method, ISO 9277:2010, Gemini 2360, Micromeritics^®^, Norcross, GA, USA), and phase purity (X’Pert PRO, Panalytical, Almelo, The Netherlands). The average particle size was determined based on the SSA and density [42]. Based on the X-ray diffraction pattern, the average size of crystallites was determined using Scherrer’s formula [42] and XRD Processor Demo application [48]. The characteristics of ZnO NPs are summarized in Table S1 (Supplementary Materials). All diffraction peaks (Figure S1—Supplementary Materials) can be well indexed to the hexagonal phase ZnO reported in JCPDS card no. 36-1451, confirming that only nano-crystalline ZnO was obtained.

### 2.3. Nanocomposite Preparation

The precise procedure of PMMA–ZnO nanocomposite preparation was described in a previously published article [25]. The main steps are described below. Thermally polymerized PMMA resin (Superacryl Plus; Spofa Dental, Jicin, Czech Republic) was used to produce the samples. The recommended mixing ratio was 22 g of powder polymer and 10 mL of liquid monomer, which represents a volume ratio of 3:1. The calculated amount of ZnO nanopowder was suspended in a liquid monomer of PMMA resin. The mixture was shaken in a Vortex VX-200 shaker (Labnet) for 10 min. and additionally sonicated for 240 s using an Elmasonic S 10/(H) (30 W; Elma Schmidbauer GmbH, Singen, Germany). Then a calculated amount of PMMA resin powder was added, so that the final 2.5%, 5%, and 7.5% mass concentration of ZnO NPs could be obtained. This was preceded by calculating the weight loss of PMMA-nanocomposite after polymerization. The experimental evaluation involved weighing each of the mixture components before a 10-min stirring and sonication process. Weight loss resulting from the release of free monomer as well as evaporation of a volatile liquid monomer while stirring and sonication of solution (based on the experiment it was designated as 4.5%). Furthermore, ZnO NPs in the solution of liquid monomer after the evaporation process increases its weight—1 g of ZnO NPs absorbs into its interior 0.1 g of monomer—which must also be taken into account. However, it is of less importance compared with weight loss during the nanocomposite preparation. Complete composition of the nanocomposites is shown in Table 1. PMMA resin without added ZnO NPs was used as a control. Originally, samples (sized 13 × 13 × 2 mm) were prepared with a model wax (Vertex Regular; Vertex-Dental BV, Zeist, The Netherlands), applying a standard procedure for the conversion of wax to PMMA resin with hard gypsum class III (Stodent; Zhermack). To obtain a smooth surface of the samples, special 0.025 mm technical foil (Divosheet; Vertex-DentalBV) was used before closing the polymerizer. The material was conventionally thermally polymerized in a polymerization integral machine (PS-2; P.E.M., Warsaw, Poland) according to the manufacturer’s instruction (gradually increasing the temperature up to 97 °C; polymerization duration in 97 °C: 30 min).

### 2.4. Technique of ZnO NPs Coating on PMMA

The precise procedure of ZnO NPs coating on PMMA was described in a previously published article [26]. Suspensions of ZnO NPs for the coating procedure were prepared in deionized water in a 0.1 wt % concentration of nanoparticles [49]. The PMMA samples (13 × 13 × 2 mm) were dipped in the prepared ZnO NPs suspension. The coating processes involved submerging an ultrasonic tip (Ti horn, 20 kHz, 80 µm amplitude, 70% efficiency, VCX750, Sonics & Materials Inc, Newtown, CT, USA) in the suspension, then due to vibration the tip induced cavitation for 5 min. The process temperature was stabilized at 30 °C ± 1 °C. The coated samples were washed with deionized water, dried, and then packaged in a laminar flow (laminar chamber, S@feflow 1.2 EuroCloneS.p.A., Pero (MI), Italy) for further research.

### 2.5. ZnO—Release Assay

Measurements of the concentration of zinc ions released to the aqueous solution were analyzed by optical emission spectrometry in inductively coupled plasma ICP-OES (spectrometer ICP-OES PerkinElmer Optima 8000, Waltham, MA, USA). This is an analytical qualitative and quantitative method, wherein the nebulizer drawn through the system as the sample solution is broken in hot plasma (6000–10,000 K) into single atoms. The excited plasma atoms or ions moving to lower energy levels emit electromagnetic waves in the field characteristics of an element used in qualitative analysis, while the quantitative study is based on the fact that the intensity of electromagnetic wave emission increases proportionally in line with the increase in the element concentration. The element concentration in the solution can be determined using internal standards and calibration curves. It is a sensitive method in which the limit of detection for most elements is within the limit of 0.1–10 ppb [50]; however, it strictly depends on the validation process. Nanocomposite samples of ZnO-PMMA sized 13 × 13 mm were divided into five groups depending on the concentration of ZnO NPs: 0% (control), 2.5%, 5%, 7.5%, and PMMA coated with ZnO-NPs (ZnO layer). Samples, at the amount of 5 per each group, were placed in extraction tubes and filled with deionized water to a final volume of 13 mL. The tubes were sealed and stored at room temperature. After 6 days, the concentration of zinc ions was measured and converted to ZnO. The data were evaluated for normal distribution using the Kolmogorov–Smirnov assay. Then, after checking the homogeneity of variance (Brown–Forsythe assay), one of the tests: Student’s *t*-test for independent samples (1) or Cochran–Cox test with separate variance estimate test (2) was performed. The level of significance was established at a *p*-value = 0.05. All data were computed using the Statistica 13.0 program (StatSoft Inc., Tulsa, OK, USA).

### 2.6. Cytotoxicity Assay

The effect of ZnO NPs on cell growth was determined in vitro with the human cell line HeLa (ECACC 93021013). The HeLa cells were cultured in DMEM medium (Gibco, San Diego, CA, USA) supplemented with 10% fetal bovine serum (Gibco, San Diego, CA, USA) essentially as previously stated [51].

For the cytotoxicity study, the ZnO NPs powder was dispersed in ultrapure water (stock solution 5 mg/mL) and sonicated for 10 min at 20 kHz. The in vitro cytotoxicity of ZnO NPs against the HeLa cell line was determined through reduction of 3-(4,5-dimethylthiazol-2-yl)-2,5-diphenyltetrazolium bromide (MTT) dye, as described previously by Paszek et al. [52]. The HeLa cells were seeded in 24-well tissue culture plates (3.5 × 10^5^ per well) and incubated using 1 mL of culture medium. After 18 h, the culture medium was replaced and the nanoparticle suspension was added to achieve their final concentrations in the wells containing cells of 0, 1, 6, 10, 20, 30, 50, and 100 mg/L. Following 24 h of incubation, 60 μL of MTT reagent (Boster Biological Technology, Pleasanton, CA, USA) was distributed into each well and the plates were further incubated for 4 h, during which time MTT was cleft into water-insoluble, purple-color formazan crystals by the mitochondrial enzyme succinate dehydrogenase of viable cells. The MTT solution was carefully removed and the formazan crystals, which had adhered to the cells, were released upon addition of DMSO and incubation at 37 °C until complete solubilization of the crystals. The absorbance of samples was measured at 540 nm (A540). The viability of the HeLa cells was expressed as follows: % viability = A540 of the samples/A540 of the control group × 100. All experiments were performed in triplicates and the results were statistically analyzed using the Student’s *t*-test for independent samples with a predetermined *p* = 0.01. Simultaneously with the MTT assay, microscopic imaging of the morphological changes in the HeLa cells treated with ZnO NPs was performed after Trypane blue staining.

## 3. Results

### 3.1. ZnO—Release Assay

**Figure 1:**
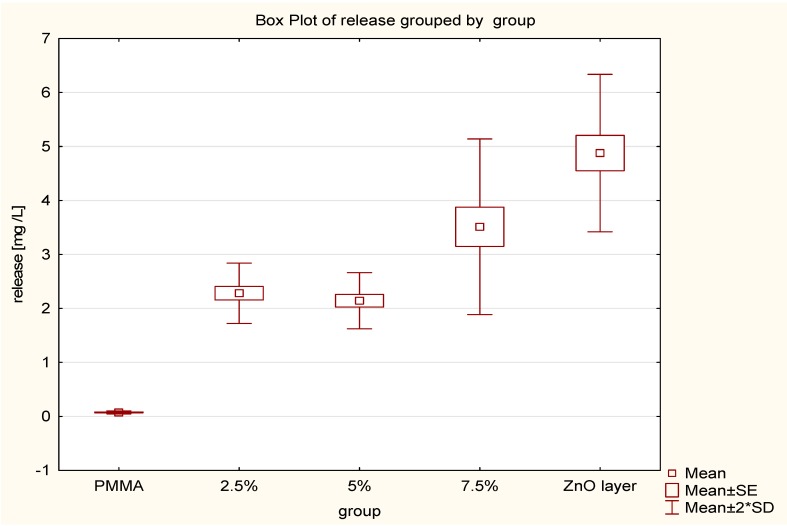
Plot of the ZnO release from nanocomposites.

The results of the statistical analysis of the ZnO NPs release to the aqueous solution are shown in Table 2 and Figure 1. The assay results were as follows: the mean of the control group was 0.074 mg/L and for the groups with the concentration of 2.5%, 5%, and 7.5% ZnO NPs were 2.281 mg/L, 2.143 mg/L, and 3.512 mg/L, respectively. The mean for the ZnO NPs layer on pure PMMA was 4.878 mg/L. No statistical significance between the 2.5% and 5% groups was obtained (*p* > 0.05). A statistical significance was achieved (*p* < 0.05) by comparing the control group to each of the test groups, and by comparing the 2.5% and 5% nanocomposites to the 7.5% nanocomposite. The maximum and minimum release of ZnO NP layers produced on pure PMMA were 4.1 mg/L and 5.649 mg/L respectively.

### 3.2. In Vitro Cytotoxicity of ZnO NPs

**Figure 2:**
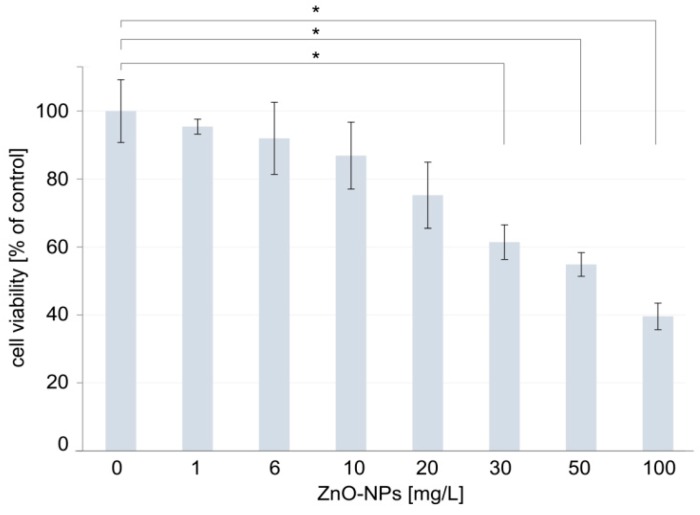
Viability of the human HeLa cells exposed to increasing concentrations of ZnO NPs. Significant differences between groups treated with ZnO NPs and the control group were marked with * (*p* < 0.01).

The cytotoxicity of the ZnO NPs was estimated on the basis of the decreased inviability of the human HeLa cells after incubation with nanoparticles with increasing concentration. There is not an established cell line model for the study of cytotoxicity of nanoparticles in dental field. There are different cell lines used in such studies, frequently not even of human origin, like NIH-3T3 mouse fibroblasts [53], L-929—mouse fibroblast cells [32], or osteogenic precursor cell from mice [54]. The HeLa cells were chosen for the study because this cell line is frequently used in cytotoxicity studies, including also these related to nanoparticles [55] and other materials used for dental purposes [56]. The viability of the HeLa cells was determined through MTT assay and the obtained results are presented in Figure 2. In the cytotoxicity study, the ZnO NPs in the concentration range of 0–100 mg/L were tested as it was in this concentration range that the cytotoxicity of ZnO NPs toward the HUVEC cells was previously tested [52]. In addition, the study took into account the concentrations of 1 mg/L and 6 mg/L, because these concentrations are just above the lowest and the highest, respectively, concentration of the nanoparticles released from the studied composites.

The results of the cytotoxicity study revealed that there were no significant differences between the cells treated with 1, 6, 10, 20 mg/L ZnO NPs and the control group after 24 h incubation (Figure 2) (*p* > 0.01). The cells’ viability at higher concentrations of nanoparticles—i.e., 30, 50, and 100 mg/L ZnO NPs—was different significantly from the control group (Figure 2) (*p* < 0.01). The study demonstrated that the relative cell viability in the presence of 30, 50, and 100 mg/L ZnO NPs amounted to 61.43 (±5.09), 54.87 (±3.48), and 39.60 (±3.91)%, respectively.

**Figure 3:**
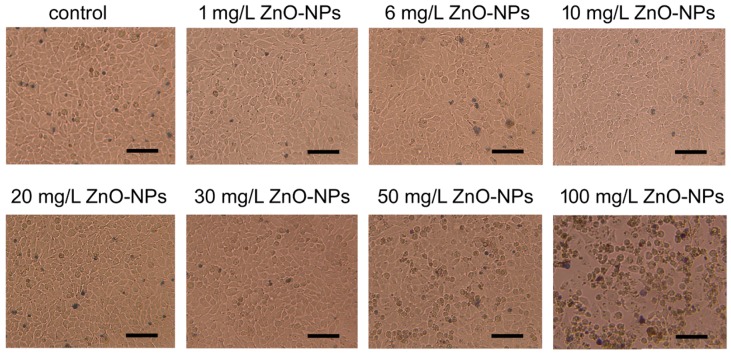
Optical microscopy images of HeLa cells treated with increasing concentration of ZnO NPs. Scale bars represent 100 μm.

Simultaneously with the MTT assay, microscopic imaging of the HeLa cells treated with ZnO NPs was performed. These microscopic observations were performed to verify the results of the MTT assay, which may be unreliable in the case of the cytotoxicity studies of some nanoparticles [57]. The microscopic observation revealed that the morphology of the cells and the structure of the cell monolayer did not differ in the control group and the cells treated with 1, 6, 10, 20, and 30 mg/L ZnO NPs (Figure 3). Morphological changes in cells were observed in the higher concentrations of ZnO NPs. In presence of 50 mg/L ZnO NPs, while the structure of the monolayer was maintained (i.e., cells adhere closely to each other covering all surface of growth without any visible gaps separating adjacent cells), the population of cells with a changed cellular shape was observed. These cells turned spherical from natively polygonal, flat cells. In the cell culture treated with 100 mg/L ZnO NPs, most of the cells turned to the spherical form and the structure of the cell monolayer were disturbed with clearly visible gaps separating adjacent cells (Figure 3).

## 4. Discussion

The objective of the study was to characterize the ZnO NPs cytotoxicity and release from PMMA–ZnO nanocomposites, as well as from the ZnO NPs layer produced on pure PMMA. The results support the rejection of the null hypothesis, stating that no differences would be found between pure PMMA and particular modifications with Zn ONPs.

The release of ZnO to the external environment (water) is an inevitable and obvious process. ZnO NPs are dispersed throughout the volume of PMMA, which means that each surface facing the outside is released from the outermost layer of the ZnO NPs. The differences between concentrations in the control and the treated samples (2.5%, 5%, and 7.5%) significantly differ from each other (*p* < 0.05). Similarly, the concentration of the released ZnO NPs in the sample at the concentration of 7.5% ZnO bacterial proteins NPs is significantly different from concentrations of nanoparticles obtained from tests of the 2.5% and 5% samples (*p* < 0.05). Statistical calculations showed no significance between the concentrations of the released ZnO NPs from 2.5% and 5% samples (*p* > 0.05). Considering the difference in the concentration by weight of ZnO NPs in the treated samples, which is 2.5%, the differences of statistical significance could be expected in concentrations between the 2.5% and 5% samples as those observed between the 7.5% and 5% samples. The specific ability of PMMA to bind some amount of ZnO NPs is a possible cause of this phenomenon. Beyond a certain limit the release of ZnO can be significantly increased, which may explain the achieved results. It should be noted that the concentration of ZnO in the surrounding environment reached the maximum value of 5.649 mg/mL for all measurements. The highest results were obtained in the ZnO NPs layer on pure PMMA, which is in line with theoretical considerations (ZnO NPs is an external layer). Notably, the previous studies evidenced that the ZnO NPs released from the 7.5% nanocomposite and the ZnO NPs layer produced on pure PMMA prevent adhesion and biofilm development by *C. Albicans* [26]. These observations indicate that the use of nanocomposites in dentistry would have a beneficial effect on the prevention of fungal infections, as long as the amount of the ZnO NPs released from them did not have a detrimental effect on the patient. To assess the possibility of safe use of the tested nanocomposites in dental applications, cytotoxicity studies of ZnO NPs were performed. First, cytotoxicity was assayed by the MTT test. The results demonstrated that the cells’ viability significantly decreased at higher concentrations of the nanoparticles, i.e., 30, 50, and 100 mg/L. These results are similar to that achieved by Paszek et al. [52], at which the cytotoxic effect on human endothelial cells of blood vessels were observed at the ZnO NPs concentration of 30 mg/L. Numerous studies indicate that the MTT assay may underestimate a compound’s cytotoxicity by overestimating the cell viability, and therefore cytotoxicity testing by the MTT assay should be supported by another assay to analyze possible toxicological effects of nanocompounds [57]. Accordingly, microscopic imaging of the HeLa cells treated with ZnO NPs was performed. The observation of morphology of the cells and the structure of the cell monolayer revealed an adverse effect of the nanoparticles in the case of the cells treated with high concentrations of ZnO NPs, i.e., 50 mg/L and 100 mg/L. Thus, the analysis of microscopic images essentially confirmed the results of the cytotoxicity studies performed by the MTT assay. Importantly, both the MTT assay and the microscope imaging did not reveal any adverse effects of nanoparticles on the cells at the concentrations of 1, 6, 10, and 20 mg/L. On the basis of the comparison of the results of ZnO NPs release and the results of the nanoparticles cytotoxicity studies, it can be concluded that the observed highest level of release of ZnO from nanomaterials (5.649 mg/mL) is more than 3 times lower than the highest concentration of ZnO NPs, in the presence of which no cytotoxic effect is observed (20 mg/L). These results indicate the possibility of safe use of the tested nanocomposites in dental applications.

The zinc oxide release from PMMA–ZnO nanocomposites assay in conjunction with testing of the cytotoxic effect of ZnO NPs suggest the safety of usage of the new biomaterial as an alternative material for denture bases. The presented results of the study, together with the previously found effect of the biomaterials on prevention of biofilm development by *C. Albicans* [26], indicate that zinc oxide released from the denture to the oral environment would induce an antifungal effect on micro-organisms settled on the mucous membrane without exerting a cytotoxic effect on the host cells.

## Figures and Tables

**Table 1:** Composition of particular nanocomposites.

Composition	2.5% Nanocomposite	5% Nanocomposite	7.5% Nanocomposite
ZnO nanopowder	0.803 g	1.605 g	2.408 g
PMMA powder polymer	22 g	22 g	22 g
Liquid monomer of PMMA	10 g	10 g	10 g

**Table 2:** Results of the ZnO release from nanocomposites: (1) Student’s *t*-test for independent samples; (2) Cochran–Cox test with separate variance estimate test.

ZnO Release *n* = 5	2.5% Mean = 2.281 mg/L	5% Mean = 2.143 mg/L	7.5% Mean = 3.512 mg/L	ZnO NPs Layer Mean = 4.878 mg/L
**0%** **Mean = 0.074 mg/L**	*t*-value = −17.6349 *p*-value = 0.0000001 (1)	*t* separ. var. est. = −17.7763 *p*-value = 0.000056 (2)	*t* separ. var. est. = −9.45474 *p*-value = 0.000696 (2)	*t* separ. var. est. = −14.7328 *p*-value = 0.000123 (2)
**2.5%** **Mean = 2.281 mg/L**		*t*-value = 0.808668 *p*-value = 0.442089 (1)	*t*-value = −3.20249 *p*-value = 0.012565 (1)	*t* separ. var. est. = −7.43784 *p*-value = 0.000608 (2)
**5%** **Mean = 2.143 mg/L**			*t*-value = −3.58721 *p*-value = 0.007113 (1)	*t* separ. var. est. = −7.90201 *p*-value = 0.000522 (2)
**7.5%** **Mean = 3.512 mg/L**				*t*-value = −2.79621 *p*-value = 0.023334 (1)

## Supplementary Materials

The following are available online at https://www.mdpi.com/2079-4991/9/9/1318/s1, Figure S1: X-ray diffraction pattern of ZnO-NPs, Table S1: Characteristics of ZnO-NPs.

## References

[1] Vance M.E., Kuiken T., Vejerano E.P., McGinnis S.P., Hochella M.F., Rejeski D., Hull M.S. (2015). Nanotechnology in the real world: Redeveloping the nanomaterial consumer products inventory. Beil. J. Nanotechnol..

[2] European Commission Nanomaterials under Biocidal Products Regulation—ECHA. https://euon.echa.europa.eu/pl/the-biocidal-products-regulation-bpr-and-nanomaterials.

[3] Jurewicz M. (2015). Biocidal Products Using Nanotechnology from a Legal Perspective. Econom. Manag..

[4] Kumar R., Umar A., Kumar G., Nalwa H.S. (2017). Antimicrobial properties of ZnO nanomaterials: A review. Ceram. Int..

[5] Sirelkhatim A., Mahmud S., Seeni A., Kaus N.H.M., Ann L.C., Bakhori S.K.M., Hasan H., Mohamad D. (2015). Review on Zinc Oxide Nanoparticles: Antibacterial Activity and Toxicity Mechanism. Nano-Micro Lett..

[6] Lallo da Silva B., Caetano B.L., Chiari-Andréo B.G., Pietro R.C.L.R., Chiavacci L.A. (2019). Increased antibacterial activity of ZnO nanoparticles: Influence of size and surface modification. Colloids Surf. B Biointerfaces.

[7] Gavrilenko E.A., Goncharova D.A., Lapin I.N., Nemoykina A.L., Svetlichnyi V.A., Aljulaih A.A., Mintcheva N., Kulinich S.A. (2019). Comparative Study of Physicochemical and Antibacterial Properties of ZnO Nanoparticles Prepared by Laser Ablation of Zn Target in Water and Air. Materials.

[8] Ali A., Phull A.R., Zia M. (2018). Elemental Zinc to Zinc nanoparticles: Is ZnO NPs crucial for life? Synthesis, toxicological and environmental concerns. Nanotechnol. Rev..

[9] Zhang Y., Nayak T.R., Hong H., Cai W. (2013). Biomedical applications of zinc oxide nanomaterials. Curr. Mol. Med..

[10] Jiang J., Pi J., Cai J. (2018). The Advancing of Zinc Oxide Nanoparticles for Biomedical Applications. Bioinorg. Chem. Appl..

[11] Zhu P., Weng Z., Li X., Liu X., Wu S., Yeung K.W.K., Wang X., Cui Z., Yang X., Chu P.K. (2016). Biomedical Applications of Functionalized ZnO Nanomaterials: From Biosensors to Bioimaging. Adv. Mater. Interfaces.

[12] Mirzaei H., Darroudi M. (2017). Zinc oxide nanoparticles: Biological synthesis and biomedical applications. Ceram. Int..

[13] Malhotra S.P., Mandal T.K. (2016). Biomedical Applications of Zinc Oxide Nanomaterials in Cancer Treatment: A review. SCIREA J. Chem..

[14] Mishra P.K., Mishra H., Ekielski A., Talegaonkar S., Vaidya B. (2017). Zinc oxide nanoparticles: A promising nanomaterial for biomedical applications. Drug Discov. Today.

[15] Bhunia A.K. (2017). ZnO Nanoparticles: Recent Biomedical Applications and Interaction with Proteins. Curr. Trends Biomed. Eng. Biosci..

[16] Bisht G., Rayamajhi S. (2016). ZnO Nanoparticles: A Promising Anticancer Agent. Nanobiomedicine.

[17] Garino N., Limongi T., Dumontel B., Canta M., Racca L., Laurenti M., Castellino M., Casu A., Falqui A., Cauda V.A. (2019). Microwave-Assisted Synthesis of Zinc Oxide Nanocrystals Finely Tuned for Biological Applications. Nanomaterials.

[18] Pokrowiecki R., Pałka K., Mielczarek A. (2018). Nanomaterials in dentistry: A cornerstone or a black box?. Nanomedicine.

[19] Martínez-Carmona M., Gun’ko Y., Vallet-Regí M. (2018). ZnO Nanostructures for Drug Delivery and Theranostic Applications. Nanomaterials.

[20] Leone F., Cataldo R., Mohamed S.S.Y., Manna L., Banchero M., Ronchetti S., Mandras N., Tullio V., Cavalli R., Onida B. (2019). Nanostructured ZnO as Multifunctional Carrier for a Green Antibacterial Drug Delivery System—A Feasibility Study. Nanomaterials.

[21] Vivek D.R. (2017). Eco-friendly and Biocompatible Acrylic Resins-A Review. J. Dental Oral Health.

[22] Bhola R., Bhola S.M., Liang H., Mishra B. (2010). Biocompatible denture polymers—A review. Trends Biomater. Artif. Organs.

[23] Vojdani M., Giti R. (2015). Polyamide as a Denture Base Material: A Literature Review. J. Dent..

[24] Craig R.G., Powers J.M. (2002). Restorative Dental Materials.

[25] Cierech M., Wojnarowicz J., Szmigiel D., Bączkowski B., Grudniak A., Wolska K., Łojkowski W., Mierzwińska-Nastalska E. (2016). Preparation and characterization of ZnO-PMMA resin nanocomposites for denture bases. Acta Bioeng. Biomech..

[26] Cierech M., Kolenda A., Grudniak A.M., Wojnarowicz J., Woźniak B., Gołaś M., Swoboda-Kopeć E., Łojkowski W., Mierzwińska-Nastalska E. (2016). Significance of polymethylmethacrylate (PMMA) modification by zinc oxide nanoparticles for fungal biofilm formation. Int. J. Pharm..

[27] Cierech M., Osica I., Kolenda A., Wojnarowicz J., Szmigiel D., Łojkowski W., Kurzydłowski K., Ariga K., Mierzwińska-Nastalska E. (2018). Mechanical and Physicochemical Properties of Newly Formed ZnO-PMMA Nanocomposites for Denture Bases. Nanomaterials.

[28] Fu S., Sun Z., Huang P., Li Y., Hu N. (2019). Some basic aspects of polymer nanocomposites: A critical review. Nano Mater. Sci..

[29] International Organization for Standardization (2015). Nanotechnologies—Vocabulary—Part 4: Nanostructured materials, ISO/TS 80004-4:2011.

[30] Anaraki M.R., Jangjoo A., Alimoradi F., Dizaj S.M., Lotfipour F. (2017). Comparison of Antifungal Properties of Acrylic Resin Reinforced with ZnO and Ag Nanoparticles. Pharm. Sci..

[31] Anwander M., Rosentritt M., Schneider-Feyrer S., Hahnel S. (2017). Biofilm formation on denture base resin including ZnO, CaO and TiO_2_ nanoparticles. J. Adv. Prosthodont..

[32] Chen R., Han Z., Huang Z., Karki J., Wang C., Zhu B., Zhang X. (2017). Antibacterial activity, cytotoxicity and mechanical behavior of nano-enhanced denture base resin with different kinds of inorganic antibacterial agents. Dent. Mater. J..

[33] Gad M.M., Fouda S.M., Al-Harbi F.A., Näpänkangas R., Raustia A. (2017). PMMA denture base material enhancement: A review of fiber, filler and nanofiller addition. Int. J. Nanomed..

[34] Kawala M., Smardz J., Adamczyk L., Grychowska N., Wieckiewicz M. (2018). Selected Applications for Current Polymers in Prosthetic Dentistry—State of the Art. Curr. Med. Chem..

[35] Niemirowicz K., Durnaś B., Piktel E., Bucki R. (2017). Development of antifungal therapies using nanomaterials. Nanomedicine.

[36] Kamonkhantikul K., Arksornnukit M., Takahashi H. (2017). Antifungal, optical and mechanical properties of polymethylmethacrylate material incorporated with silanized zinc oxide nanoparticles. Int. J. Nanomed..

[37] Kamonkhantikul K. (2017). Effect of 3-methacryloxypropyltrimethoxysilane Modified Zinc Oxide Nanoparticles Incorporated in Polymethylmethacrylate Material on Antifungal, Optical and Mechanical Properties. Ph.D. Thesis.

[38] Popovic P., Bobovnik R., Bolka S., Vukadinovic M., Lazic V., Rudolf R. (2017). Synthesis of PMMA/ZnO nanoparticles composite used for resin teeth. Mater. Tech..

[39] Salahuddin N., El-Kemary M., Ibrahim E. (2018). Reinforcement of polymethyl methacrylate denture base resin with ZnO nanostructures. Int. J. Appl. Ceram. Technol..

[40] Raj I., Mozetic M., Jayachandran V., Jose J., Thomas S., Kalarikkal N. (2018). Fracture resistant, Antibiofilm adherent, self-assembled PMMA/ZnO nanoformulations for Biomedical applications: Physico-chemical and biological perspectives of nano reinforcement. Nanotechnology.

[41] Kati F.A. (2019). Effect of the incorporation of zinc oxide nanoparticles on the flexural strength of auto- polymerized acrylic resins. J. Oral. Res..

[42] Wojnarowicz J., Opalinska A., Chudoba T., Gierlotka S., Mukhovskyi R., Pietrzykowska E., Sobczak K., Lojkowski W. (2016). Effect of water content in ethylene glycol solvent on the size of ZnO nanoparticles prepared using microwave solvothermal synthesis. J. Nanomater..

[43] Wojnarowicz J., Chudoba T., Koltsov I., Gierlotka S., Dworakowska S., Lojkowski W. (2018). Size control mechanism of ZnO nanoparticles obtained in microwave solvothermal synthesis. Nanotechnology.

[44] Wojnarowicz J., Chudoba T., Gierlotka S., Lojkowski W. (2018). Effect of Microwave Radiation Power on the Size of Aggregates of ZnO NPs Prepared Using Microwave Solvothermal Synthesis. Nanomaterials.

[45] Wojnarowicz J., Chudoba T., Gierlotka S., Sobczak K., Lojkowski W. (2018). Size Control of Cobalt-Doped ZnO Nanoparticles Obtained in Microwave Solvothermal Synthesis. Crystals.

[46] Majcher A., Wiejak J., Przybylski J., Chudoba T., Wojnarowicz J. (2013). A novel reactor for microwave hydrothermal scale-up nanopowder synthesis. Int. J. Chem. React. Eng..

[47] Dąbrowska S., Chudoba T., Wojnarowicz J., Łojkowski W. (2018). Current Trends in the Development of Microwave Reactors for the Synthesis of Nanomaterials in Laboratories and Industries: A Review. Crystals.

[48] Nanopowder XRD Processor Demo. http://science24.com/xrd/.

[49] Woźniak B., Dąbrowska S., Wojnarowicz J., Chudoba T., Łojkowski W. (2017). Coating synthetic materials with zinc oxide nanoparticles acting as a UV filter. Glass Ceram..

[50] Szczepaniak W. (1999). Instrumental Methods of Chemical Analysis.

[51] Wiśniewski J., Krawczyk-Balska A., Bielecki J. (2006). Associated roles of hemolysin and p60 protein for the intracellular growth of Bacillus subtilis. FEMS Immunol. Med. Microbiol..

[52] Paszek E., Czyz J., Woźniacka O., Jakubiak D., Wojnarowicz J., Łojkowski W., Stępień E. (2012). Zinc oxide nanoparticles impair the integrity of human umbilical vein endothelial cell monolayer in vitro. J. Biomed. Nanotechnol..

[53] Acosta-Torres L.S., Mendieta I., Nuñez-Anita R.E., Cajero-Juárez M., Castaño V.M. (2012). Cytocompatible antifungal acrylic resin containing silver nanoparticles for dentures. Int. J. Nanomed..

[54] Shen X.T., Zhang Y.Z., Xiao F., Zhu J., Zheng X.D. (2017). Effects on cytotoxicity and antibacterial properties of the incorporations of silver nanoparticles into the surface coating of dental alloys. J. Zhejiang Univ. Sci. B.

[55] Venkatesan J., Singh S.K., Anil S., Kim S.-K., Shim M.S. (2018). Preparation, Characterization and Biological Applications of Biosynthesized Silver Nanoparticles with Chitosan-Fucoidan Coating. Molecules.

[56] Bozzini B., Barca A., Bogani F., Boniardi M., Carlino P., Mele C., Verri T., Romano A. (2014). Electrodeposition of nanostructured bioactive hydroxyapatite-heparin composite coatings on titanium for dental implant applications. J. Mater. Sci. Mater. Med..

[57] Nuñez-Anita R.E., Acosta-Torres L.S., Vilar-Pineda J., Martínez-Espinosa J.C., de la Fuente-Hernández J., Castaño V.M. (2014). Toxicology of antimicrobial nanoparticles for prosthetic devices. Int. J. Nanomed..

