# Supplementary material for: Zinc Oxide Nanoparticles Cytotoxicity and Release from Newly Formed PMMA–ZnO Nanocomposites Designed for Denture Bases
Source: Nanomaterials (Basel). 2019 Sep 15;9(9):1318. doi: 10.3390/nano9091318 (PMC6781076; doi:10.3390/nano9091318)
Supplement: Supplementary file 1 [file nanomaterials-09-01318-s001.pdf]

nanomaterials

MDPI

Supplementary Materials

Zinc Oxide Nanoparticles Cytotoxicity and Release from Newly Formed PMMA–ZnO Nanocomposites Designed for Denture Bases

Mariusz Cierech [1,*], Jacek Wojnarowicz [2], Adam Kolenda [1], Agata Krawczyk-Balska [3], Emilia Prochwicz [3], Bartosz Woźniak [2], Witold Łojkowski [2] and Elżbieta Mierzwińska-Nastalska [1]

[1] Department of Prosthodontics, Medical University of Warsaw, Warsaw 02-006, Poland
[2] Institute of High Pressure Physics, Polish Academy of Sciences, Warsaw 01-142, Poland
[3] Department of Applied Microbiology, Biological and Chemical Research Centre, Faculty of Biology, University of Warsaw, Warsaw 02-089, Poland
* Correspondence: mariusz.cierech@wp.pl; Tel.: +48-22-502-18-86

Table S1. Characteristics of ZnO-NPs.

Sample	Specific surface area (SSA), $a_s \pm \sigma$ (m²/g)	Skeleton density, $\varrho_s \pm \sigma$ (g/cm³)	Average particle size from SSA BET, d±σ (nm)	Average crystalite size, Scherer's formula (nm)	Average grain size, Nanopowder XRD Processor Demo, d ± σ (nm)
ZnO-NPs	39.8 ± 0.2	5.17 ± 0.05	29	27 ± 2	26 ± 8

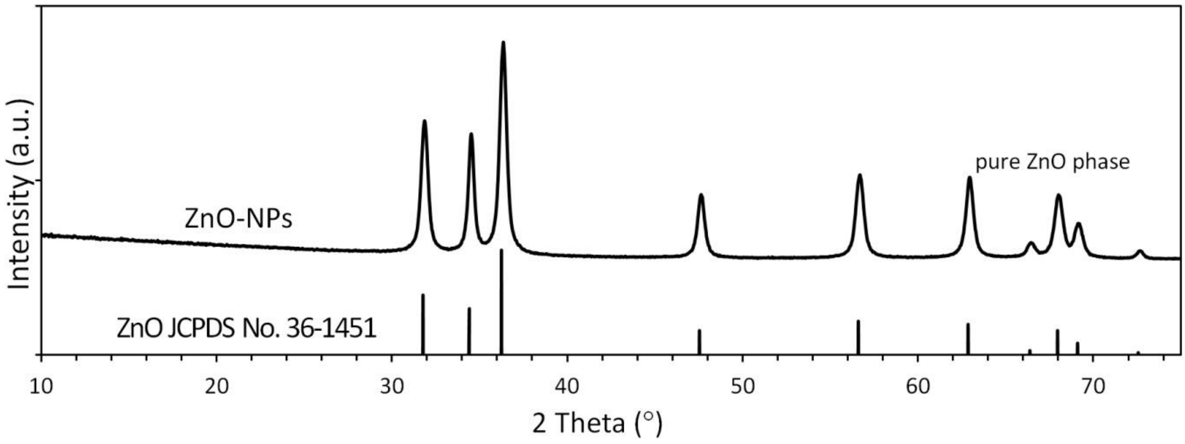

Figure S1. X-ray diffraction pattern of ZnO-NPs.
